# Survival Outcomes in Metastatic Germ Cell Tumors: A Multicenter Study from Turkey

**DOI:** 10.3390/medicina61060951

**Published:** 2025-05-22

**Authors:** Gul Sema Yildiran Keskin, Ozge Yetginoglu, Sertac Vurgun, Evrican Zin Guzel, Fariz Emrah Ozkan, Mesut Yilmaz, Cem Murat Soylemez, Yesim Agyol, Sinem Akbas, Muhammed Fatih Sagiroglu, Gizem Yildirim, Huseyin Salih Semiz, Ali Murat Tatli, Ferhat Ekinci, Ramazan Cosar, Ramazan Acar, Musa Baris Aykan, Ismail Erturk, Nuri Karadurmus

**Affiliations:** 1 Department of Medical Oncology, Health Science University, Gulhane Research and Training Hospital, Ankara 06010, Turkey; 2 Department of Medical Oncology, Dokuz Eylül University School of Medicine, Izmir 35210, Turkey; ozyetginoglu@gmail.com (O.Y.);; 3 Department of Medical Oncology, Akdeniz University School of Medicine, Antalya 07058, Turkey; sertac@live.com (S.V.);; 4 Department of Medical Oncology, Manisa Celal Bayar University School of Medicine, Manisa 45030, Turkey; evricanzin@gmail.com (E.Z.G.); drferhatekinci@hotmail.com (F.E.); 5 Department of Medical Oncology, Afyonkarahisar Health Sciences University, Afyonkarahisar 03200, Turkey; dr.emrah.oz@gmail.com (F.E.O.); ramazancosar@gmail.com (R.C.); 6 Department of Medical Oncology, Sakarya Research and Training Hospital, Sakarya 54100, Turkey; mstsnm08@gmail.com; 7 Department of Medical Oncology, Izmir City Hospital, İzmir 35540, Turkey; cm.soylemez@yahoo.com; 8 Department of Medical Oncology, Marmara University School of Medicine, Istanbul 34854, Turkey; yesimagyol@gmail.com; 9 Department of Medical Oncology, Koc University Hospital, Istanbul 34010, Turkey; sinem_kocak@yahoo.com; 10 Department of Medical Oncology, Gaziantep City Hospital, Gaziantep 27470, Turkey; 11 Department of Medical Oncology, Health Science University, Gulhane School of Medicine, Ankara 06010, Turkey; gizemyildirim_@hotmail.com (G.Y.); musabarisaykan@gmail.com (M.B.A.);; 12 Department of Medical Oncology, Ufuk University School of Medicine, Ankara 06510, Turkey; dr_racar@yahoo.com

**Keywords:** testicular cancer, metastatic germ cell tumor, chemotherapy, IGCCCG, seminoma, non-seminoma

## Abstract

*Background/Objectives*: Metastatic testicular germ cell tumor (mGCT) is a highly curable disease with first-line cisplatin-based combination chemotherapy. This study aims to evaluate the clinicopathological characteristics and survival outcomes of patients with metastatic testicular cancer in a nationwide multicenter cohort. *Methods*: This multicenter retrospective cohort study included 316 male patients diagnosed with mGCT who were treated with first-line cisplatin-based chemotherapy across 10 institutions in Turkey between 2011 and 2024. Clinical and pathological data, including International Germ Cell Cancer Collaborative Group (IGCCCG) risk classification, treatment details, and survival outcomes, were analyzed. *Results*: The median age of the cohort was 28 years, and 76.3% of patients were diagnosed with non-seminoma. According to IGCCCG risk stratification, 53.2% had good-risk, 25.3% intermediate-risk, and 21.5% poor-risk disease. Median follow-up was 38.4 months. Among patients with seminoma, the 5-year overall survival (OS) rate was 100% in the good-risk group and 87.5% in the intermediate-risk group. In patients with non-seminoma, 5-year OS rates were 96.6%, 86.9%, and 65.1% in the good-, intermediate-, and poor-risk groups, respectively. Among 125 patients who received salvage treatment, high-dose chemotherapy (HDCT) significantly improved survival in the International Prognostic Factors Study Group (IPFSG) very high-risk group (3-year OS: 55.0% vs. 16.3% with conventional-dose chemotherapy (CDCT), *p* = 0.007). *Conclusions*: This study provides the first large-scale nationwide dataset on mGCT outcomes in Turkey, demonstrating overall survival rates comparable to international cohorts. The findings emphasize the importance of a multidisciplinary approach, adherence to treatment guidelines, and optimal surgical interventions in improving patient outcomes.

## 1. Introduction

Testicular cancer (TC) is a rare neoplasm, accounting for only 1% of tumors in males. However, it is the most common malignant solid tumor among men aged 15–40 years. According to GLOBOCAN 2022, the global incidence of testicular cancer is 1.7 per 100,000 per year, with a remarkably low mortality rate of 0.21 per 100,000 per year [1].

Patients with de novo metastatic testicular germ cell tumors (GCTs) account for approximately 30% of all GCT cases [2]. Furthermore, 15–30% of patients with clinical stage I (CSI), characterized by disease confined to the testis without radiological or biochemical evidence of metastasis, relapse during active surveillance [3]. It is a highly curable disease with first-line cisplatin-based combination chemotherapy, even in advanced stages [4]. Although significant advancements in oncology have been made over the past two decades, the triplet chemotherapy regimen of bleomycin, etoposide, and cisplatin (BEP) has remained the backbone first-line treatment for metastatic testicular cancer since 1987 [5].

The International Germ Cell Cancer Collaborative Group (IGCCCG) classification is a prognostic model for metastatic GCT based on the identification of independent adverse clinical factors, such as primary tumor site, metastatic sites, and levels of serum tumor markers [6]. This system stratifies patients into good-, intermediate-, and poor-risk groups, guiding treatment decisions and survival estimations. The IGCCCG Update Consortium has recently revised survival outcomes for metastatic GCTs. The reported 5-year overall survival (OS) rates were 96% in good-, 89% in intermediate-, 67% in poor-risk patients with metastatic non-seminoma [7], and 95% in good- and 88% in intermediate-risk patients with metastatic seminoma [8]. Although the standard of care has remained the same for years, improved survival rates have been achieved through a multidisciplinary approach in experienced centers, timely surgical interventions, effective salvage and supportive therapies, and close patient follow-up [9].

Recent national and regional studies have underscored the relevance of country-specific data in understanding survival trends. For instance, a Japanese cohort of 138 patients with metastatic non-seminoma reported a 5-year overall survival (OS) of 82% in the poor-risk group, suggesting favorable outcomes even in advanced disease when managed in experienced centers [10]. Meanwhile, a Latin American multicenter study of 482 patients showed 5-year OS rates of 94.3%, 83.5%, and 65.1% in the good-, intermediate-, and poor-risk groups, respectively [11]. These findings illustrate how survival outcomes may vary across regions, possibly due to differences in healthcare infrastructure, referral systems, and access to multidisciplinary expertise. This emphasizes the need for national data to inform context-specific clinical strategies.

Therefore, this study aims to evaluate the 5-year OS and progression-free survival (PFS) of patients with metastatic GCTs who received first-line cisplatin-based combination chemotherapy, according to the IGCCCG prognostic classification. Additionally, it seeks to provide an international comparison of survival outcomes for this population-based cohort in Turkey.

## 2. Methods

This was a multicenter retrospective cohort study. A total of 316 male patients who received first-line cisplatin-based combination chemotherapy for metastatic GCT at 10 institutions in Turkey between 2011 and 2024 were identified. Staging was performed using a combination of serum tumor markers—alpha-fetoprotein (AFP), human chorionic gonadotropin (hCG), and lactate dehydrogenase (LDH)—and computed tomography of the chest, abdomen, and pelvis, in accordance with the American Joint Committee on Cancer (AJCC) TNM Staging Classification for Testicular Cancer 8th edition (2017).

Inclusion criteria were as follows: (1) histologically confirmed mGCT; (2) age ≥ 18 years; (3) adequate clinical, imaging, and tumor marker data for IGCCCG-based risk classification; (4) treatment with first-line cisplatin-based combination chemotherapy at participating centers; and (5) at least one year follow-up period. Patients with missing data regarding tumor stage, treatment modalities, or inadequate follow-up duration were excluded from this study.

Clinical characteristics, including age at diagnosis, primary tumor location, histology, metastatic sites, and serum levels of AFP, HCG, and LDH, were extracted from electronic medical records. Risk stratification was performed using the IGCCCG classification, which considers three key prognostic factors: primary tumor site (testis/retroperitoneum vs. mediastinum), presence of non-pulmonary visceral metastases (NPVM), and serum tumor marker levels prior to the initiation of chemotherapy. Patients were classified into good-, intermediate-, or poor-risk groups accordingly. For patients who received salvage treatment, risk assessment was based on the International Prognostic Factors Study Group (IPFSG) model, incorporating five variables: primary tumor site, response to first-line chemotherapy, progression-free interval, serum tumor marker levels at relapse, and metastatic sites at relapse. Patients were stratified into five prognostic categories: very low, low, intermediate, high, and very high risk. Additionally, we recorded the type and duration of chemotherapy, and details of subsequent treatments, including interventions for residual disease and salvage therapies in cases of recurrence.

Treatment response was assessed using a combination of radiologic imaging (based on RECIST version 1.1) and serum tumor marker kinetics. Complete response (CR) was defined as the disappearance of all detectable lesions and the normalization of serum tumor markers (STMs). Partial response (PR) required a ≥30% reduction in lesion size, with or without normalization of tumor markers. It was further categorized into STM-negative PR and STM-positive PR due to its prognostic relevance. Stable disease (SD) referred to the absence of sufficient change to meet the criteria for CR or PD, while progressive disease (PD) was defined as ≥20% increase in lesion size or emergence of new lesions, with or without rising tumor markers.

This multicenter study was approved by the Ethics Committee of Gulhane Training and Research Hospital (approval number: 2025-170; date of approval: 11 March 2025).

## 3. Statistical Analysis

All statistical analyses were performed by using SPSS software version 25.0 (IBM Corp., Armonk, NY, USA). Descriptive statistics were reported as frequencies and percentages for categorical variables, and as medians with interquartile ranges (IQR) for continuous variables. Median follow-up duration was estimated using the reverse Kaplan–Meier method. PFS was defined as the time from the initiation of chemotherapy for metastatic disease to disease progression, relapse, or death. OS was calculated from the start of the chemotherapy to the last follow-up or death from any cause. The probabilities of PFS and OS was estimated using the Kaplan–Meier method, and differences between subgroups of patients were assessed with the log-rank test. A *p*-value < 0.05 was considered statistically significant in all analyses.

## 4. Results

### 4.1. Patient Characteristics

The median age of the 316 patients was 28 years (interquartile range [IQR]23–36), with 56 patients (17.7%) aged over 40 years. Seminoma and non-seminoma were diagnosed in 75 (23.7%) and 241 (76.3%) patients, respectively. A primary gonadal GCT was identified in 293 patients (92.7%). The most common histological subtype was mixed germ cell tumor (63.9%). Regarding the IGCCC prognostic group classification, 53.2% of patients were classified into the good prognosis group, while 25.3% and 21.5% were classified into the intermediate and poor prognosis groups, respectively. The rate of relapse from clinical stage I (CSI) disease was 10.4%. Non-pulmonary visceral metastases (NPVMs) were present in 56 patients (17.7%), while 119 patients (37.7%) had pulmonary metastases. Platinum resistance, defined as disease relapse or progression within three months after completing a platinum-containing regimen, was observed in 57 patients (18%). Patient characteristics stratified by seminoma and non-seminoma subgroups are presented in Table 1.

### 4.2. First-Line Treatment and Response

The type of first-line chemotherapy and the number of cycles administered are shown in Table 2. The majority of patients received three or four cycles of BEP (90%). A favorable response to first-line therapy, defined as the normalization of serum tumor markers (STM) with or without residual radiologic lesions, was observed in 78% of patients. Among these, 38.1% achieved a complete response, characterized by STM normalization and the absence of residual masses. Retroperitoneal lymph node dissection (RPLND) for residual disease was performed in 64 patients (20.3%). Additionally, 13 patients received radiotherapy and 9 underwent metastasectomy. In total, 75 patients underwent surgery after chemotherapy. Pathological examination revealed viable cancer in 28 patients and they required two additional cycles of chemotherapy after surgery. Pathology was consistent with necrosis or fibrosis in 30 patients and teratoma in 15 patients.

### 4.3. Salvage Treatment

A total of 125 patients received second-line salvage therapy. Fifteen of them received salvage treatment despite the absence of documented disease progression due to residual disease that were not amenable to local therapies. Among those who received second-line treatment, high-dose chemotherapy (HDCT) with carboplatin and etoposide (CE) was the most commonly chosen regimen (79 patients). Additionally, 46 patients received conventional-dose chemotherapy (CDCT), with paclitaxel, ifosfamide, and cisplatin (TIP) being the most frequently administered second-line CDCT regimen. Details of second-line and subsequent treatments, along with additional therapies, are presented in Table 2.

### 4.4. Survival Analysis

The median follow-up period was 38.4 months (95% confidence interval [CI], 34.5 to 42.1 months). In the overall study population, the estimated median mPFS was 133.4 months (95% CI: −/−), and mOS had not yet been reached at the time of analysis, as the majority of patients were still alive. The 5-year PFS rate for the entire cohort was 62.6%, and the 5-year OS rate was 87.5%.

**Figure 1:**
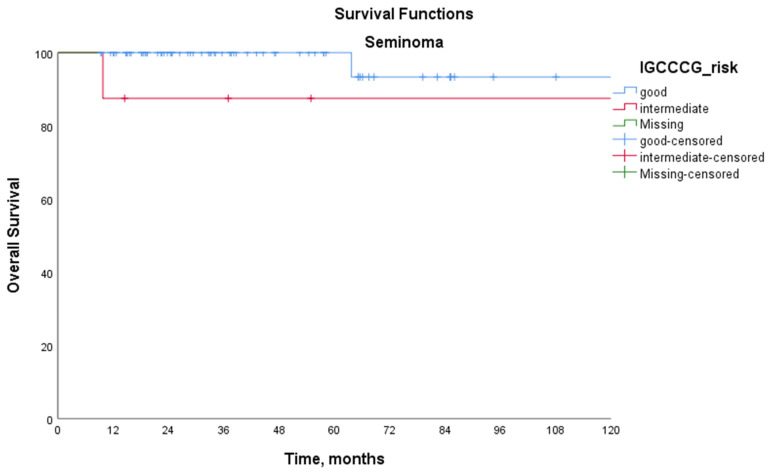
Kaplan–Meier overall survival curves for patients with seminoma stratified by the International Germ Cell Cancer Collaborative Group (IGCCCG) prognostic classification. The 5-year overall survival (OS) rates were 100% in the good-risk group and 87.5% in the intermediate-risk group (log-rank test, *p* = 0.186).

**Figure 2:**
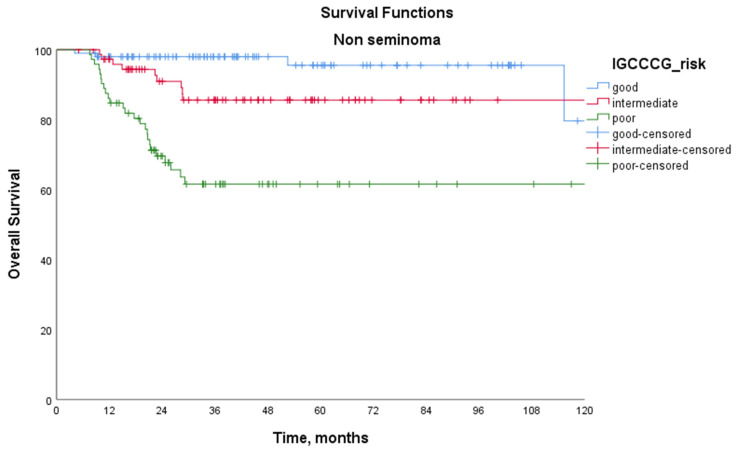
Kaplan–Meier overall survival curves for patients with non-seminoma according to the International Germ Cell Cancer Collaborative Group (IGCCCG) prognostic classification. The 5-year overall survival (OS) rates were 96.6% in the good-risk group, 86.9% in the intermediate-risk group, and 65.1% in the poor-risk group (log-rank test: *p* = 0.046 for good vs. intermediate, *p* < 0.001 for good vs. poor, and *p* = 0.001 for intermediate vs. poor).

Table 3 presents the survival outcomes according to the IGCCCG prognostic group classification. Among patients with seminoma, the 5-year PFS was 82.8% in the IGCCCG good-risk group and 46.9% in the intermediate-risk group (*p* = 0.030). For patients with non-seminoma, the 5-year PFS rates were 80.9% in the good-risk group, 51.9% in the intermediate-risk group, and 30.9% in the poor-risk group (*p* < 0.001 for good vs. intermediate, *p* < 0.001 for good vs. poor, and *p* = 0.001 for intermediate vs. poor). Among patients with seminoma, the 5-year OS rates were 100% in the good-risk group and 87.5% in the intermediate-risk group (*p* = 0.186). For patients with non-seminoma, the 5-year OS rates were 96.6%, 86.9%, and 65.1% in the good-, intermediate-, and poor-risk groups, respectively (*p* = 0.046 for good vs. intermediate, *p* < 0.001 for good vs. poor, and *p* = 0.001 for intermediate vs. poor). Kaplan–Meier survival curves according to the IGCCCG prognostic classification are shown in Figure 1 for patients with seminoma and Figure 2 for patients with non-seminoma.

**Figure 3:**
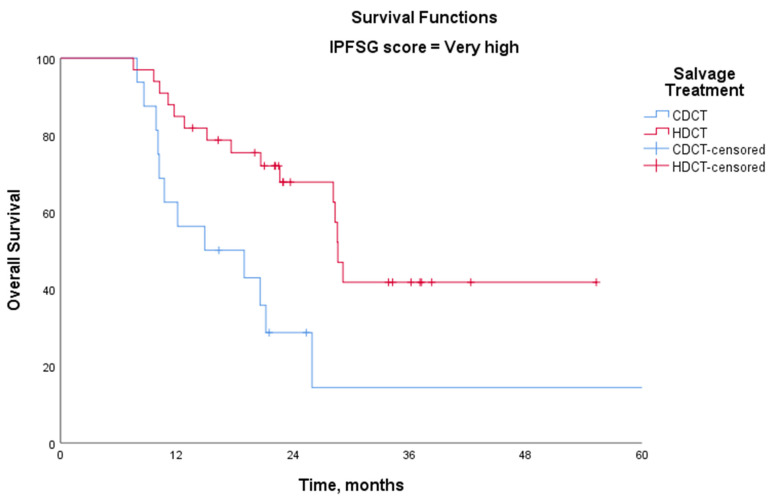
Kaplan–Meier overall survival curves for relapsed/refractory patients with IPFSG very high-risk scores, comparing high-dose chemotherapy (HDCT; 3-year OS: 55.0%) and conventional-dose chemotherapy (CDCT; 3-year OS: 16.3%) (log-rank test, *p* = 0.007).

For relapsed/recurrent patients, salvage treatment outcomes based on the IPFSG score are presented in Table 4. Except for the very high-risk group, there was no significant difference between CDCT and HDCT. However, in the very high-risk group, the 3-year OS rate was significantly higher with HDCT (55.0%) compared with CDCT (16.3%) (*p* = 0.007) (Figure 3).

## 5. Discussion

In this multicenter retrospective study, we analyzed the clinical characteristics and 5-year survival outcomes of patients treated for metastatic GCTs. According to the IGCCCG prognostic classification, the 5-year OS rates in the seminoma subgroup were 100% in the good-risk group and 87.5% in the intermediate-risk group. For non-seminoma, the 5-year OS rates were 96.6%, 86.9%, and 65.1% in the good-, intermediate-, and poor-risk groups, respectively. This study represents the first large-scale dataset from Turkey, and its survival outcomes are consistent with the latest IGCCCG update, reflecting an improving trend in treatment success.

Survival rates for metastatic testicular cancer are known to be higher in specialized medical centers compared with global averages. In 2018, Indiana University updated and published their 5-year OS results, reporting 97% in the good-risk group, 92% in the intermediate-risk group, and 73% in the poor-risk group. This success has been attributed to their multidisciplinary clinical approach and the implementation of structured initial treatment plans in experienced centers [9]. In a Japanese cohort study that included 138 patients with metastatic non-seminoma, the 5-year PFS rate was 64%, while the 5-year OS rate reached 82%, even in the poor-risk group. Notably, for patients treated between 2000 and 2018 in this Japanese cohort, the 5-year OS improved to 85% for the poor-risk group. As observed at Indiana University, this success was linked to the early referral of poor-prognosis patients to specialized centers and the cumulative expertise of these institutions [10].

In the study by Diogo A. Bastos et al., conducted by the Latin American Cooperative Oncology Group (LACOG) and including 482 patients with advanced germ cell tumors, the 5-year OS rates were 94.3% in the good-risk group, 83.5% in the intermediate-risk group, and 65.1% in the poor-risk group [11]. Similarly, a North American population-based cohort study reported 5-year OS rates of 94%, 87%, and 65% for patients with metastatic non-seminoma in the good-, intermediate-, and poor-risk groups, respectively [12]. These findings further support the consistency of survival outcomes observed in our study and reinforce the effectiveness of standardized treatment approaches across different populations. Standard first-line chemotherapy for metastatic GCTs has remained unchanged for decades. Despite significant improvements in overall survival over the years, progression-free survival has shown little change. The GETUG 13 trial, a prospective randomized phase 3 study, explored whether dose-dense therapy could improve survival outcomes in patients with suboptimal response to first-line BEP. After one BEP cycle, patients with an unfavorable STM decline were randomized to continue either BEP (Unfav-BEP group) or a dose-dense regimen (Unfav-dose-dense group), incorporating two cycles of paclitaxel-BEP-oxaliplatin and two cycles of platin-ifosfamide-bleomycin, GCSF supported chemotherapy [13]. At long-term follow-up, 5-year PFS was 58.9% in the dose-intensive arm and 46.7% in the standard BEP arm (HR, 0.65; 95% CI, 0.44–0.97; *p* = 0.036). There was no statistical difference in 5-year OS (70.9% vs. 61.3%, *p* = 0.22). Although dose-intensive therapies seem to be a reasonable strategy to prolong PFS in high-risk patients, increased toxicity, especially neuropathy, should be considered. Furthermore, it is important to note that these intensified regimens may reduce the need for HDCT in the salvage setting [14].

In this study, we also evaluated salvage treatment strategies in relapsed/refractory cases. Among 125 patients, 79 received induction TIP followed by HDCT with autologous stem cell transplantation (ASCT) in the case of progression, whereas 46 patients received CDCT, primarily four cycles of TIP. When comparing CDCT vs. HDCT, no significant difference in OS was observed, except in the very high-risk group according to the IPFSG classification. In this subgroup, the 3-year OS was significantly higher with HDCT (55%) compared with CDCT (16.3%, *p* = 0.007). These findings align with previous evidence suggesting that HDCT may offer a survival advantage in patients with very high-risk disease. The TIGER trial (NCT02375204), which directly compares CDCT (TIP) vs. HDCT (TI-CE), is expected to provide further insights into the optimal salvage treatment approach.

Our study also highlights challenges in managing poor-risk patients, particularly regarding treatment compliance and surgical management. PFS rates were notably lower in the poor-risk subgroup, which could be attributed to treatment delays, suboptimal compliance, or variations in clinical practice. Additionally, inadequate surgical resection of residual disease may have played a role. Of 164 patients with a partial response, only 64 (39%) underwent RPLND. Given that post-chemotherapy retroperitoneal masses ≥1 cm in non-seminoma tumors often harbor viable cancer [15], RPLND is essential for optimal disease control [16,17]. However, due to its potential complications, such as retrograde ejaculation and infertility, RPLND should be performed at high-volume centers by experienced surgeons [16].

In parallel with therapeutic advances, efforts have also focused on improving diagnostic accuracy. Although classic serum tumor markers are widely used, their sensitivity and specificity are limited, particularly in subtypes such as pure seminoma or teratoma, which makes accurate disease assessment challenging [18]. In this context, microRNAs (miRNAs), especially miR-371a-3p, have shown high diagnostic performance, with reported sensitivity of 84–89% and specificity of 90–99%. Moreover, serum levels of miR-371a-3p have been associated with tumor burden, treatment response, and early relapse detection [19]. Although our study did not evaluate molecular biomarkers, miRNA-based approaches may further enhance diagnostic accuracy and facilitate personalized follow-up strategies in patients with metastatic GCTs.

This study has several limitations. First, its retrospective design may introduce selection bias. Second, although it is a multicenter study, the cohort size is smaller compared with international studies. Third, the median follow-up period is relatively short, limiting the ability to assess long-term outcomes. Nevertheless, this study reflects clinical experience from a broad range of institutions, including non-referral centers, and offers valuable insight into treatment outcomes in routine practice across the country.

In conclusion, this study provides real-world evidence on the survival outcomes of metastatic germ cell tumors in Turkey. While overall survival rates are comparable to international cohorts, further improvements may be achieved by enhancing treatment adherence, fostering multidisciplinary collaboration, and ensuring optimal surgical management, particularly when delivered in high-volume experienced centers. These strategies are especially important for improving outcomes in high-risk patients.

## Figures and Tables

**Table 1:** Patients and disease baseline characteristics.

Baseline Characteristics	All Cohort (N = 316) N (%)	Seminoma (N = 75) N (%)	Non-Seminoma (N = 241) N (%)
Median age, years (IQR)	28 (23–36)		
Under 40	260 (82.3)	51 (68)	209 (86.4)
Over 40	56 (17.7)	24 (32)	32 (13.3)
Location of primary tumor			
Gonadal	293 (92.7)	72 (96)	221 (91.7)
Retroperitoneal	13 (4.1)	0 (0)	13 (5.4)
Mediastinal	10 (3.2)	3 (4)	7 (2.9)
Tumor histology			
Pure seminoma	75 (23.7)	75 (100)	
Non-seminoma	241 (76.3)		241 (100)
Mixed germ cell tumors	201 (63.6)		201(83.4)
Embrional carcinoma	26 (8.2)		26 (10.8)
Yolksac	7 (2.2)		7 (2.9)
Teratoma	5 (1.6)		5 (2.1)
Choriocarcinoma	2 (0.6)		2 (0.8)
IGCCCG risk at diagnosis			
Good	168 (53.2)	67 (89.3)	101 (41.9)
Intermediate	80 (25.3)	8 (10.7)	72 (29.9)
Poor	68 (21.5)		68 (28.2)
Stage at metastatic diagnosis			
IIA	42 (13.3)	14 (18.7)	28 (11.6)
IIB	52 (16.5)	18 (24)	34 (14.1)
IIC	56 (17.7)	22 (29.3)	34 (14.1)
IIIA	57 (18.0)	8 (10.7)	49 (20.3)
IIIB	42 (13.3)	8 (10.7)	34 (14.1)
IIIC	67 (21.2)	5 (6.7)	62 (25.7)
Metastatic sites			
Lymph nodes/retroperitoneal	304 (96.2)	74 (98.7)	230 (95.4)
Pulmonary	119 (37.7)	7 (9.3)	112 (46.5)
NPVM	56 (17.7)	7 (9.3)	49 (20.3)
Liver	37 (11.7)	3 (4)	34 (14.1)
Brain	8 (2.5)	1 (1.3)	7 (2.9)
Bone	19 (6.0)	3 (4)	16 (6.6)
Postorchiectomy median AFP ng/mL (IQR)	15 (3.3–344)	-	
AFP level < 1000	238 (79.1)		167 (72.6)
AFP level 1000–10,000	42 (14)		42 (18.3)
AFP level ≥ 10,000	21 (7)		21 (9.1)
Postorchiectomy median hCG mlU/mL (IQR)	16 (1–505)		
hCG level < 5000	257 (87.7)	67 (98.5)	190 (84.4)
hCG level 5000–50,000	17 (5.8)	1 (1.5)	16 (7.1)
hCG level ≥ 50,000	19 (6.5)		19 (8.4)
Postorchiectomy median LDH	306 (200–543)		
<2.5 × ULN	217 (72.1)	55 (77.5)	162 (70.4)
>2.5 × ULN	84 (27.9)	16 (22.5)	68 (29.6)
Relapse from Stage I	33 (10.4)	16 (21.3)	17 (7.1)
Platinum resistance (PFI < 3 months)	57 (18)	6 (8)	51 (21.1)
Progression after first-line treatment	110 (34.8)	15 (20)	95 (39.4)
IPFSG Risk score for salvage treatment	125 (39.6)	15 (20)	110 (45.6)
Very low	7 (2.2)	7 (9.3)	0 (0)
Low	22 (7)	4 (5.3)	18 (7.5)
Intermediate	23 (7.3)	2 (2.7)	21(8.7)
High	30 (9.5)	1 (1.3)	29 (12)
Very high	42 (13.3)	1 (1.3)	41 (17)
Unknown	1 (0.3)		1 (0.4)
Progression after second-line treatment	44 (13.9)	1 (1.3)	43 (17.8)
Exitus	34 (10.8)	2 (2.7)	32 (13.3)

Abbreviations: IQR—interquartile range; IGCCCG—International Germ Cell Cancer Collaborative Group; NPVM—non-pulmonary visceral metastasis; AFP—alpha fetoprotein; hCG—human chorionic gonadotropin; LDH—lactate dehydrogenase; PFI—platinum-free interval; IPFSG—International Prognostic Factors Study Group.

**Table 2:** Treatment characteristics.

Treatment Profile	No of Patients (%)
Metastatic first-line chemotherapy	316 (100)
3 × BEP	133 (42.1)
4 × BEP	120 (38)
4 × BEP + 2 × EP	22 (7)
4 × EP	14 (4.4)
4 × VIP	10 (3.2)
4 × BEP + 2 × VIP	5 (1.6)
3 × BEP + 1 × EP	4 (1.3)
3 × EP	4 (1.3)
6 × EP	2 (0.6)
3 × TIP	1 (0.3)
4 × VIP + 2 × EP	1 (0.3)
Responses after first-line treatment	
Complete response	123 (38.1)
Partial response	
STM (−)	129 (39.9)
STM (+)	35 (10.8)
Stable disease	12 (3.7)
Progressive disease	27 (7.4)
Additional treatment after first-line	
RPLND	64 (20.3)
Radiotherapy	13 (4.1)
Metastasectomy	9 (2.8)
Chemotherapy	28 (8.9)
Pathology after additional surgery	
Viable Tumor	28 (8.9)
Necrosis/Fibrosis	30 (9.5)
Teratoma	15 (4.7)
Metastatic second-line (salvage) treatment	125 (39.6)
CDCT	46 (14.6)
4 × TIP	33 (10.4)
4 × VIP	7 (2.2)
GemPOX	4 (1.3)
VeIP	2 (0.6)
HDCT	79 (25)
Responses after salvage treatment	
Complete response	32 (10.1)
Partial response	
STM (−)	72 (22.8)
STM (+)	7 (2.2)
Stable disease	5 (1.6)
Progressive disease	9 (2.8)
Additional treatment after second-line	
RPLND	9 (2.8)
Radiotherapy	10 (3.2)
Metastasectomy	7 (2.2)
Maintanence oral etoposide	28 (8.9)
Third-line and beyond treatment	
GemPOX/GemOX	24 (7.6)
HDCT	6 (1.9)
TIP	2 (0.6)
Brentuximab	2 (0.6)

Abbreviations: BEP—bleomycin, etoposide, and cisplatin; EP—etoposide and cisplatin; VIP—etoposide, ifosfamide, and cisplatin; TIP—paclitaxel, ifosfamide, and cisplatin; STM—serum tumor marker; VeIP—vinblastine, ifosfamide, and cisplatin; GemPOX—gemcitabine, paclitaxel, and oxaliplatin; RPLND—retroperitoneal lymph node dissection; CDCT—conventional-dose chemotherapy; HDCT—high-dose chemotherapy.

**Table 3:** Survival outcomes according to the IGCCCG prognostic group classification.

		IGCCCG Prognostic Group			
		Good	Intermediate	Poor	*p*
Seminoma	5-year PFS (%)	82.8	46.9	-	0.030
	5-year OS (%)	100	87.5	-	0.186
Non-seminoma	5-year PFS (%)	80.9	51.9	30.9	<0.001
					<0.001
					0.001
	5-year OS (%)	96.6	86.9	65.1	0.046
					<0.001
					0.001
All cohort	5-year PFS (%)	81.4	51.3	30.9	<0.001
					<0.001
					0.001
	5-year OS (%)	97.7	87.1	65.1	0.017
					<0.001
					0.001

*p* values for the good/intermediate, good/poor, and intermediate/poor comparisons. IGCCCG—International Germ Cell Cancer Collaborative Group; OS—overall survival; PFS—progression-free survival.

**Table 4:** Three-year OS rates of salvage treatment according to IPFSG score.

IPFSG Score	Salvage	N. of Patients	N. of Events	3 Year OS %	*p*
Very low	CDCT	5	0	-	-
	HDCT	2	0	-	
Low	CDCT	8	0	-	-
	HDCT	14	0	-	
Intermediate	CDCT	8	2	87.5	0.570
	HDCT	15	3	84.8	
High	CDCT	10	3	61.7	0.371
	HDCT	20	5	83.4	
Very high	CDCT	14	10	16.3	0.007
	HDCT	28	10	55.0	

Abbreviations: IPFSG—International Prognostic Factors Study Group; CDCT—conventional-dose chemotherapy; HDCT—high-dose chemotherapy; OS—overall survival.

## Data Availability

The data that support the findings of this study are not publicly available due to privacy reasons but are available from the corresponding author upon reasonable request.
